# Neutralizing antibody levels associated with injectable and aerosolized Ad5-nCoV boosters and BA.2 infection

**DOI:** 10.1186/s12916-023-02942-3

**Published:** 2023-07-03

**Authors:** Fuzhen Wang, Baoying Huang, Yao Deng, Shaobai Zhang, Xiaoqiang Liu, Lei Wang, Qianqian Liu, Li Zhao, Lin Tang, Wenling Wang, Xiaoqi Wang, Fei Ye, Weijun Hu, Haitao Yang, Siquan Wang, Jiao Ren, Xiaoyu Liu, Cangning Wang, Xuhua Guan, Ruize Wang, Yan Zheng, Xianfeng Zhang, Hui Zheng, Dan Wu, Zhijie An, Wenbo Xu, Lawrence E. Rodewald, George F. Gao, Zundong Yin, Wenjie Tan

**Affiliations:** 1 National Immunization Program, Chinese Center for Disease Control and Prevention, Beijing, China; 2 National Health Commission (NHC) Key Laboratory of Biosafety, Institute for Viral Disease Control and Prevention, Chinese Center for Disease Control and Prevention, Beijing, China; 3 Shaanxi Provincial Center for Disease Control and Prevention, Xi’an, China; 4 Yunnan Provincial Center for Disease Control and Prevention, Kunming, China; 5 Hubei Provincial Center for Disease Control and Prevention, Wuhan, China; 6 Chinese Center for Disease Control and Prevention, Beijing, China

**Keywords:** COVID-19 vaccine, Booster immunization, Aerosolized Ad5-nCoV vaccine, Neutralizing antibody, BA.5

## Abstract

**Background:**

Several COVID-19 vaccines are in widespread use in China. Few data exist on comparative immunogenicity of different COVID-19 vaccines given as booster doses. We aimed to assess neutralizing antibody levels raised by injectable and inhaled aerosolized recombinant adenovirus type 5 (Ad5)-vectored COVID-19 vaccine as a heterologous booster after an inactivated COVID-19 vaccine two-dose primary series.

**Methods:**

Using an open-label prospective cohort design, we recruited 136 individuals who had received inactivated vaccine primary series followed by either injectable or inhaled Ad5-vectored vaccine and measured neutralizing antibody titers against ancestral SARS-CoV-2 virus and Omicron BA.1 and BA.5 variants. We also measured neutralizing antibody levels in convalescent sera from 39 patients who recovered from Omicron BA.2 infection.

**Results:**

Six months after primary series vaccination, neutralizing immunity against ancestral SARS-CoV-2 was low and neutralizing immunity against Omicron (B.1.1.529) was lower. Boosting with Ad5-vectored vaccines induced a high immune response against ancestral SARS-CoV-2. Neutralizing responses against Omicron BA.5 were ≥ 80% lower than against ancestral SARS-CoV-2 in sera from prime-boost subjects and in convalescent sera from survivors of Omicron BA.2 infection. Inhaled aerosolized Ad5-vectored vaccine was associated with greater neutralizing titers than injectable Ad5-vectored vaccine against ancestral and Omicron SARS-CoV-2 variants.

**Conclusions:**

These findings support the current strategy of heterologous boosting with injectable or inhaled Ad5-vectored SARS-CoV-2 vaccination of individuals primed with inactivated COVID-19 vaccine.

**Supplementary Information:**

The online version contains supplementary material available at 10.1186/s12916-023-02942-3.

## Background

The ongoing COVID-19 pandemic has caused emergence of SARS-CoV-2 variants [1]. Omicron BA.1 was discovered in late 2021 and quickly replaced Delta to become the dominant variant, characterized by high transmissibility and immune escape [2–4]. Subsequently, several Omicron sub-lineages gradually become prevalent SARS-CoV-2 variants, including BA.2, which appeared in early March 2022 [5, 6], and BA.4 and BA.5, which are anticipated to become globally dominant variants due to their transmissibility and replacement of earlier variants [7].

Safe and effective COVID-19 vaccines are critically important for responding to the pandemic and reducing morbidity and mortality from SARS-CoV-2 infection [8, 9]. However, many reports have shown waning of vaccine-elicited neutralizing antibodies in both magnitude and protective efficacy for multiple types of vaccines, especially against SARS-CoV-2 variants [10–14]. A third homologous booster dose is known to have a satisfactory safety profile and a high immune response [15–17]. Heterologous boosting induces higher neutralizing responses than does homologous prime-boost vaccination [18–20].

In October 2021, China launched a booster dose campaign among those who had completed a primary series with either two doses of inactivated vaccines or one dose of recombinant adenovirus type 5 (Ad5)-vectored vaccine at least 6 months earlier. By January 2023, more than 820 million people in mainland China received booster doses.

Inactivated whole-virion SARS-CoV-2 vaccines, such as BBIBP-CorV and CoronaVac, have been used in large-scale vaccination programs in China as both primary series and booster doses. Injectable and inhaled versions of the recombinant Ad5-vectored vaccine, Convidecia (injectable) and Convidecia Air (inhaled) by CanSinoBIO, which were shown to be safe and immunogenic in clinical trials [21–23], have been authorized as booster doses in China. Clinical trials and real-world studies have shown that following inactivated vaccine primary series, homologous inactivated and heterologous adenovirus-vectored, protein subunit vaccines, and mRNA vaccines boosters all enhance immune response [19, 24–28]. These data are providing evidence for boosting strategies. It is not known how the neutralizing antibody response of a booster dose of inhaled aerosolized Ad5-vectored vaccine compares with an injectable Ad5-vectored vaccine booster dose.

We conducted a prospective cohort study of healthy adults aged 18–59 years that assessed neutralizing antibody responses against ancestral virus and Omicron subvariants (BA.1 and BA.5) of two COVID-19 vaccines as booster doses 6 months after two doses of inactivated vaccines (BBIBP-CorV or CoronaVac) and of convalescent sera from survivors of Omicron BA.2 infection. We report results of our study.

## Methods

### Study design setting

The design was a prospective cohort, open-label study of booster vaccination against COVID-19 and was conducted from August 2021 to September 2022 in Beijing municipality and Yunnan and Shaanxi provinces, China.

### Participants

Participants were eligible if they were 18–59 years old and had received two dose of inactivated COVID-19 vaccines (BBIBP-CorV, Sinopharm Beijing CNBG; or CoronaVac, Sinovac,Co., Ltd) at least 6 months before enrolment. Participant exclusion criteria were history of infection with SARS-CoV-2, history of using blood products or immunosuppressive drugs after primary doses, and history of serious vaccine-related adverse reaction. Exit and suspension criteria were leaving the local area or becoming lost to follow-up, subject request to withdraw or suspend the survey, and failure to complete follow-up and sample collection due to serious adverse reactions or health conditions.

In addition to sera from the vaccinated subjects, we obtained convalescent sera following a COVID-19 outbreak in Shaanxi province. On March, 2022, Xian City reported a COVID-19 case, leading to an outbreak involving Xian, Baoji, and Hanzhong cities. All cases were infected with the SARS-CoV-2 Omicron (B.1.1.529) BA.2 variant; 5.1% of infections were asymptomatic, and the 94.9% of cases that were symptomatic were of mild or moderate severity. Convalescent sera were obtained from cases in this outbreak for comparison with sera from vaccinated subjects in our study.

### Procedures

Provincial centers for disease control and prevention (CDCs) identified potential subjects using contact and vaccination data from their immunization information systems and screened for willingness to participate and eligibility via telephone. Individuals who met all inclusion and no exclusion criteria were invited to a baseline visit during which they signed informed consent to participate. The baseline visit was considered day 0 and was timed to be in the window of booster dose eligibility for the study. During the baseline visit, we administered a questionnaire to obtain demographic data, assigned subjects to study groups, drew blood, and administered booster doses. Participants received their day 0 booster dose at least 6 months after having completed primary vaccination.

Injectable Convidecia was administered via injection in the deltoid muscle; aerosolized Convidecia was administered orally by inhalation. Inhaled aerosolized Ad5-nCoV vaccine, Convidecia Air, was developed by Institute of Biotechnology (Beijing, China) and CanSino Biologics (Tianjin, China) and was supplied as a liquid formulation of 1.5 mL per vial at a concentration of 1 × 10^11^ viral particles per milliliter. We used a continuous vaporing system to aerosolize the Ad5-nCoV and flow the aerosolized vaccine into a disposable cup. Participants inhaled 0.1 mL of the aerosolized vaccine droplets through their mouth.

Participants were divided into three groups according to the type of priming and booster vaccines: BBIBP-CorV + Convidecia (group A), BBIBP-CorV + aerosolized Convidecia (group B), CoronaVac + aerosolized Convidecia (group C). Blood samples were obtained for neutralizing antibody analyses at baseline (immediately prior to the booster dose, day 0) and on day 7 and month 6 following booster dose administration.

In Shaanxi province, we recruited subjects who recovered from COVID-19 in the outbreak and obtained demographic information and blood samples 4 to 24 days after each person’s first positive polymerase-chain-reaction (PCR) assay during their infection with the Omicron BA.2 variant.

### Laboratory testing

Neutralization assays were conducted in a BSL-3 laboratory. Serum nAb responses were assessed by reduction of cytopathic effect (CPE) in Vero cells with infectious SARS-CoV-2 strain 19nCoV-CDC-Tan-HB01 (HB01), 19nCoV-CDC-Tan-Omicron-BA.1-SH01 (BA.1), and 19nCoV-CDC-Tan-Omicron-BA.5-SH01 (BA.5). Briefly, serum was inactivated at 56 °C for 30 min and successively diluted from 1:4 to the required concentration in 2-fold series. An equal volume of challenge virus solution containing 100 CCID50 virus was added. After neutralization in a 37 °C incubator for 2 h, a 1.5–2.5 × 10^5^/ml cell suspension was added to the wells; cytopathic effect was assessed 4 days after infection. Neutralization titers (NT_50_) were expressed as the reciprocal of the highest dilution protecting 50% of the cells from the virus challenge. To facilitate comparison of SARS-CoV-2 neutralization assay data from multiple assay formats and vaccines, we used the WHO international standard (IS) and an internal neutralization standard.

### Statistical analysis

We used mean ± standard deviations (SD), medians, and interquartile ranges (IQR) for continuous variables and numbers (percentages) for categorical variables. Immunogenicity was expressed by nAb seroconversion percentage, geometric mean titers (GMT), with associated 95% confidence intervals (CI). Antibody titers were log-transformed to calculate GMT per group. We used Kruskal-Wallis test to compare GMTs of convalescent sera with GMTs of sera from the prime-booster group. All analyses were conducted using SAS statistical software (version 9.4; SAS Institute Inc., Cary, NC, USA) and R (version 4.1.0). Statistical tests were two-sided, and we considered *P* values of 0.05 or less as statistically significant.

## Results

### Participants

**Fig. 1:**
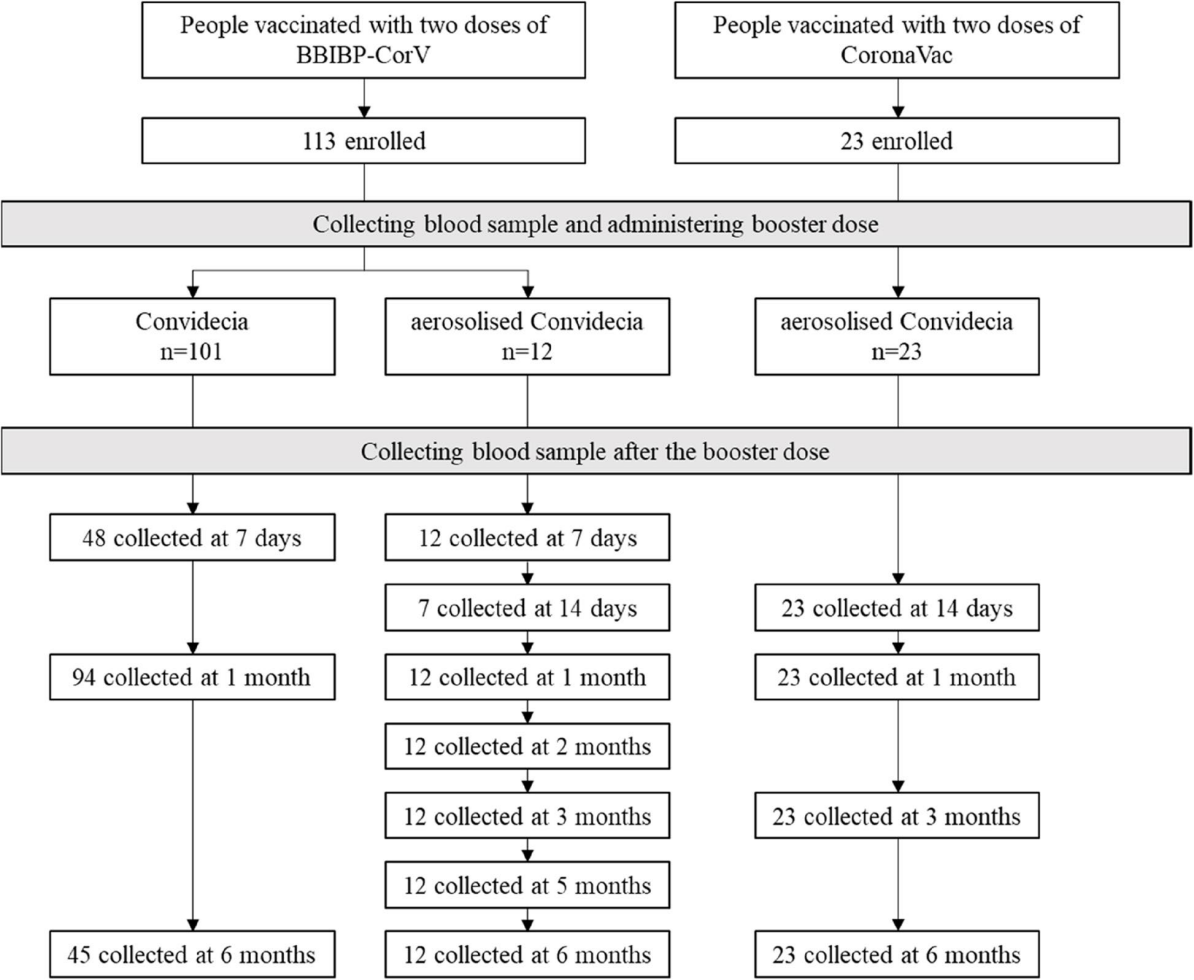
Study flow diagram

Between August 2021 and September 2022, we recruited 136 individuals who met all inclusion criteria and no exclusion criteria and who had completed full primary series COVID-19 vaccination 6 months before enrollment (Fig 1). Participants ranged in age from 19 to 59 years, with a mean age of 37.5 (SD 10.3 years); 47.1% of participants were male; 29.4% were overweight (24 ≤ BMI < 28.0 kg/m^2^) and 5.2% were obese (BMI ≥ 28.0 kg/m^2^); 11.0% had ≥ 1 underlying medical conditions; 101 were in group A: BBIBP-CorV + injectable Convidecia, 12 were in group B: BBIBP-CorV + aerosolized Convidecia, and 23 were in group C: CoronaVac + aerosolized Convidecia; the median interval between completing primary vaccination and receiving booster doses was 188 days (range: 184–191) in group A, 328 days (180–550) in group B, and 204 days (185–261) in group C (Table 1).

**Table 1:** Baseline characteristics by prime-boost COVID-19 vaccine study group

Characteristic	**Group A**: BBIBP-CorV+ Convidecia (*n* = 101)	**Group B**: BBIBP-CorV+ aerosolized Convidecia (*n* = 12)	**Group C**: CoronaVac+ aerosolized Convidecia (*n* = 23)
Gender			
Male	47 (46.53)	3 (25.00)	14 (60.87)
Female	54 (53.47)	9 (75.00)	9 (39.13)
Age group (at first dose, years)			
18–29	26 (25.74)	6 (50.00)	4 (17.39)
30–39	36 (35.64)	3 (25.00)	6 (26.09)
40–49	24 (23.76)	3 (25.00)	7 (30.43)
50–59	15 (14.86)	0 (0)	6 (26.09)
BMI (kg/m^2^)			
Normal (BMI< 24)	66 (65.35)	12 (100)	11 (47.83)
Overweight (24≤BMI< 28)	28 (27.72)	0 (0)	12 (52.17)
Obese (BMI ≥ 28)	7 (6.93)	0 (0)	0 (0)
Underlying comorbidities			
≥ 1 type	7 (6.93)	0 (0)	8 (34.78)
None	94 (93.07)	12 (100)	15 (65.22)
Interval between first and second doses (days)			
21–27	94 (93.07)	11 (91.67)	19 (82.61)
28–	7 (6.93)	1 (8.33)	4 (17.39)
Interval between primary and booster doses (days)			
180–194 (6–6.5 months)	101 (100)	1 (8.33)	2 (8.7)
195–209 (6.5–7 months)	0 (0)	0 (0)	19 (82.61)
240–299 (8–10 months)	0 (0)	2 (16.67)	2 (8.7)
300–359 (10–12 months)	0 (0)	8 (66.67)	0 (0)
360– (12– months)	0 (0)	1 (8.33)	0 (0)

We obtained 39 blood samples from recovered COVID-19 patients who had been infected with the SARS-CorV-2 Omicron BA.2 variant. These survivors ranged in age from 18 to 59 years, with a mean age of 41.7 (SD 12.0 years); 18 (46.2%) were male and 21 (53.88%) were female; 15 (38.5%) were unvaccinated and 24 (61.5%) completed primary vaccination with two doses of inactivated vaccine more than 6 months before their infection. Among vaccinated subjects, intervals between their last dose of vaccine and their Omicron infection ranged from 188 to 274 days, with a median of 261.5 days. We considered these vaccinated and Omicron-infected subjects to have hybrid immunity.

### NAb response before and after booster vaccination

Six months after primary vaccination, NT50 positivity rates were 64.5%, 24.3%, and 4.3% against ancestral virus, BA.1, and BA.5, respectively, and corresponding nAb titers were 4.7 (4.1–5.4), 2.5 (2.3–2.8), and 2.1 (2.0–2.2). Ten months after primary vaccination, NT50 positivity rates were 25.0%, 0%, and 0% against ancestral virus, BA.1, and BA.5, and corresponding nAb titers were 2.6 (1.1–6.3), 2.0, and 2.0.

**Fig. 2:**
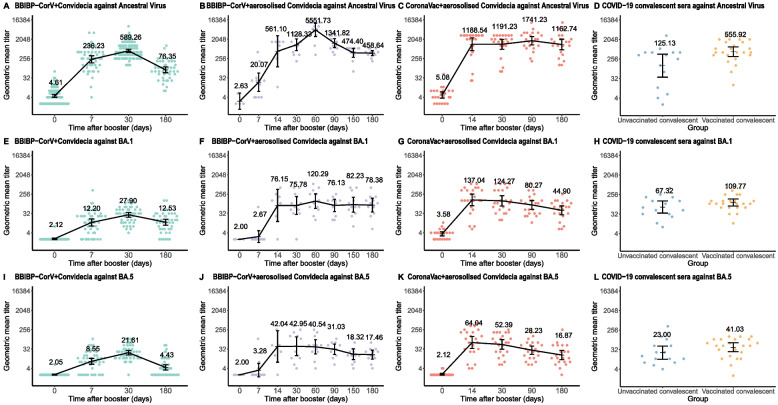
Immune responses against ancestral virus, BA.1, and BA.5 by booster vaccine and convalescent sera. GMTs against ancestral virus **A**, **B**, **C**, BA.1 **E**, **F**, **G**, and BA.5 **I**, **J**, **K** were showed over time by prime-boost groups. GMTs of unvaccinated convalescent and vaccinated convalescent against ancestral virus **D**, BA.1 **H**, and BA.5 **L** were also showed. Comparing Nab response between natural infection, vaccination, and hybrid immunity: against ancestral virus—group A vs. natural immunity: *P* = 0.08; group A vs. hybrid immunity: *P* = 0.99; group B vs. natural immunity:* P* = 0.04; group B vs. hybrid immunity: *P* = 0.52; group C vs. natural immunity:* P* = 0.004; group C vs. hybrid immunity: *P* = 0.06; against BA.1—group A vs. natural immunity: *P* = 0.08; group A vs. hybrid immunity: *P* < 0.001; group B vs. natural immunity:* P* = 0.99; group B vs. hybrid immunity: *P* = 0.91; group C vs. natural immunity: *P* = 0.71; group C vs. hybrid immunity: *P* = 1.00; against BA.5—group A vs. natural immunity: *P* = 1.00; group A vs. hybrid immunity: *P* = 0.04; group B vs. natural immunity:* P* = 0.83; group B vs. hybrid immunity: *P* = 1.00; group C vs. natural immunity: *P* = 0.27; group C vs. hybrid immunity: *P* = 0.93

Figure 2 and Table 2 show NT_50_ positivity rates and geometric mean titers (GMTs) of neutralizing antibodies against ancestral virus, BA.1, and BA.5 by boosting group before (day 0) and 7 days–6-months after boosting.

**Table 2:** Immune responses against ancestral virus, BA.1, and BA.5 by booster vaccine and convalescent sera

Participants	Group	Sample collection time	Ancestral			BA.1			BA.5			Comparing	
			*n*	Positive rate (%)	Geometric mean titers (1:) GMT (95% CI)	*n*	Positive rate (%)	Geometric mean titers (1:) GMT (95% CI)	*n*	Positive rate (%)	Geometric mean titers (1:) GMT (95% CI)	BA.5 vs. ancestral declining percentage	BA.5 vs. BA.1 declining percentage
Prime-booster group	A: BBIBP-CorV + Convidecia	0 day	101	62.38	4.61 (3.92, 5.43)	47	4.26	2.12 (1.95, 2.31)	47	2.13	2.05 (1.95, 2.15)	55.60%	3.50%
		7 days	48	100	236.23 (160.84, 346.95)	47	78.72	12.20 (8.35, 17.82)	47	74.47	8.55 (6.26, 11.69)	96.38%	29.91%
		1 month	94	100	589.26 (497.26, 698.27)	47	100	27.90 (21.58, 36.07)	47	97.87	21.61 (17.06, 27.36)	96.33%	22.54%
		6 months	45	100	76.35 (55.47, 105.09)	45	86.67	12.53 (9.06, 17.33)	45	53.33	4.43 (3.31, 5.92)	94.20%	64.65%
	B: BBIBP-CorV + aerosolized Convidecia	0 day	4	25.00	2.63 (1.1, 6.31)	4	0	2	4	0	2	24.02%	0.00%
		7 days	12	100	20.07 (7.5, 53.71)	12	8.33	2.67 (1.41, 5.04)	12	25.00	3.28 (1.59, 6.8)	83.64%	− 23.01%
		14 days	7	100	561.10 (104.49, 3013.13)	7	85.71	76.15 (13.57, 427.13)	7	85.71	42.04 (7.75, 228.15)	92.51%	44.80%
		1 month	12	100	1128.33 (598.32, 2127.83)	12	91.67	75.78 (29.13, 197.11)	12	100	42.95 (15.78, 116.93)	96.19%	43.32%
		2 months	12	100	5551.73 (2723.38, 11317.47)	12	100	120.29 (55.39, 261.23)	12	100	40.54 (18.91, 86.92)	99.27%	66.30%
		3 months	12	100	1341.82 (832.66, 2162.32)	11	100	76.13 (39.3, 147.49)	11	100	31.03 (17.82, 54.02)	97.69%	59.25%
		5 months	12	100	474.4 (293.47, 766.89)	12	100	82.23 (36.82, 183.68)	12	100	18.32 (10.28, 32.63)	96.14%	77.73%
		6 months	12	100	458.64 (348.89, 602.93)	12	100	78.38 (37.72, 162.9)	12	100	17.46 (10.78, 28.27)	96.19%	77.73%
	C: CoronaVac + aerosolized Convidecia	0 day	23	73.91	5.08 (3.63, 7.12)	23	65.22	3.58 (2.87, 4.47)	23	8.7	2.12 (1.95, 2.32)	58.18%	40.70%
		14 days	23	100	1188.54 (600.03, 2354.24)	23	100	137.04 (73.5, 255.5)	23	100	64.04 (33.58, 122.14)	94.61%	53.27%
		1 month	23	100	1191.23 (696.52, 2037.33)	23	100	124.27 (71.52, 215.93)	23	100	52.39 (31.28, 87.77)	95.60%	57.84%
		3 months	22	100	1741.23 (1110.25, 2730.82)	22	100	80.27 (49.78, 129.44)	22	100	28.23 (18.21, 43.75)	98.38%	64.83%
		6 months	21	100	1162.74 (657.21, 2057.14)	21	100	44.90 (27.82, 72.48)	21	95.24	16.87 (10.16, 28.01)	98.55%	62.43%
COVID-19 convalescent sera	Unvaccinated	15 days after infection	15	93.33	125.13 (36.71, 426.49)	15	100	67.32 (35.34, 128.25)	15	100	23.00 (11.40, 46.40)	81.62%	65.83%
	Fully vaccinated with 2 doses inactivated vaccine	15 days after infection	24	100	555.92 (332.52, 929.42)	24	100	109.77 (75.72, 159.13)	24	95.83	41.03 (25.44, 66.17)	92.67%	62.62%

All COVID-19 vaccines given as booster doses induced significantly higher immunogenicity post boost compared with before the boost. In group A (BBIBP-CorV + injectable Convidecia), at 7 days post-boosting, GMTs increased to 236.2 (160.8–347.0), 12.2 (8.4–17.8), and 8.6 (6.3–11.7) against ancestral virus, BA.1, and BA.5. At 1 month, GMTs increased to 589.3 (497.3–698.3), 27.9 (21.6–36.1), and 21.6 (17.1–27.4) against ancestral virus, BA.1, and BA.5. At 6 months, GMTs were 76.4 (55.5–105.1), 12.5 (9.1–17.3), and 4.4 (3.3–5.9) against ancestral virus, BA.1, and BA.5.

In group B (BBIBP-CorV + aerosolized Convidecia), at 7 days post-boosting, GMTs increased to 20.1 (7.5–53.7), 2.7 (1.4–5.0), and 3.3 (1.6–6.8) against ancestral virus, BA.1, and BA.5. At 14 days, GMTs increased to 561.1 (104.5–3013.1), 76.2 (13.6–427.1), and 42.0 (7.8–228.2). At 1 month, GMTs increased to 1128.3 (598.3–2127.8), 75.8 (29.1–197.1), and 43.0 (15.8–116.9). At 2 months, GMTs were 5551.7 (2723.4–11317.5), 120.3 (55.4–261.2), and 40.5 (18.9–86.9). At 3 months, GMTs were 1341.8 (832.7–2162.3), 76.1 (39.3–147.5), and 31.0 (17.8–54.0). At 5 months, GMTs were 474.4 (293.5–766.9), 82.2 (36.8–183.7), and 18.3 (10.3–32.6). At 6 months, GMTs were 458.6 (348.9–602.9), 78.4 (37.7–162.9), and 17.5 (10.8–28.3).

In group C (CoronaVac + aerosolized Convidecia), at 14 days post-boosting, GMTs increased to 1188.5 (600.0–2354.2), 137.0 (73.5–255.5), and 64.0 (33.6–122.1) against ancestral virus, BA.1, and BA.5. At 1 month, GMTs increased to 1191.2 (696.5–2037.3), 124.3 (71.5–215.9), and 52.4 (31.3–87.8). At 3 months, GMTs were 1741.2 (1110.3–2730.8), 80.3 (49.8–129.4), and 28.2 (18.2–43.8). At 6 months, GMTs were 1162.7 (657.2–2057.1), 44.9 (27.8–72.5), and 16.9 (10.2–28.0).

### Comparing nAb response to ancestral virus, BA.1, and BA.5

Compared with nAb response to ancestral virus, the neutralizing response to BA.5 was a statistically significant > 80% lower. Neutralizing response to BA.5 was also lower than to BA.1.

In group A (BBIBP-CorV + Convidecia), at 1 month post-boosting, the GMT against BA.5 was 96.3% lower than against ancestral virus and 22.5% lower than against BA.1. On day 180 post-boosting, the GMT against BA.5 was 94.2% lower than against ancestral virus and 64.7% lower than against BA.1.

In group B (BBIBP-CorV + aerosolized Convidecia), at 1 month post-boosting, the GMT against BA.5 was 96.2% lower than against ancestral virus and 43.3% lower than against BA.1. On day 180 post-boosting, the GMT against BA.5 was 96.2% lower than against ancestral virus and 77.7% lower than against BA.1.

In group C (CoronaVac + aerosolized Convidecia), at 1 month post-boosting, the GMT against BA.5 was 95.6% lower than against ancestral virus and 57.8% lower than against BA.1. On day 180 post-boosting, the GMT against BA.5 was 98.6% lower than against ancestral virus and 62.4% lower than against BA.1.

### Comparing Nab response between natural infection, vaccination, and hybrid immunity

Table 2 shows NT50 positivity rates and GMTs of neutralizing antibodies of convalescent sera against ancestral virus and Omicron variant by vaccination history. Respective neutralizing antibodies against ancestral virus, BA.1, and BA.5 were 125.1 (36.7–426.5), 67.3 (35.3–128.3), and 23.0 (11.4–46.4) in unvaccinated patients and 555.9 (332.5–929.4), 109.8 (75.7–159.1), and 41.0 (25.4–66.2) in vaccinated patients.

At 1 month post-boosting, GMTs against ancestral virus following boosting with injectable Convidecia or aerosolized Convidecia were comparable to GMTs of patients with hybrid immunity (group A vs. hybrid immunity: *P* = 0.99; group B vs. hybrid immunity: *P* = 0.52; group C vs. hybrid immunity: *P* = 0.06), with 1.1-fold (group A), 2.0-fold (group B), and 2.1-fold (group C) greater than that of hybrid immunity.

Against BA.1 or BA.5, GMTs following boosting with aerosolized Convidecia were comparable to GMTs of patients with hybrid immunity, and GMT following boosting with injectable Convidecia was comparable to patients with natural immunity (against BA.1: group A vs. natural immunity: *P* = 0.08; group A vs. hybrid immunity: *P* < 0.001; group B vs. natural immunity:* P* = 0.99; group B vs. hybrid immunity: *P* = 0.91; group C vs. natural immunity: *P* = 0.71; group C vs. hybrid immunity: *P* = 1.00. Against BA.5: group A vs. natural immunity: *P* = 1.00; group A vs. hybrid immunity: *P* = 0.04; group B vs. natural immunity:* P* = 0.83; group B vs. hybrid immunity: *P* = 1.00; group C vs. natural immunity: *P* = 0.27; group C vs. hybrid immunity: *P* = 0.93).

Neutralizing antibody levels by study group and gender and by study group and BMI group are in Supplementary Information (see Additional file 1: Tables S1-S2).

## Discussion

Our cohort study measured neutralizing antibody responses from injectable and inhaled aerosolized Ad5-vectored COVID-19 vaccine in 136 participants who had completed primary series with inactivated COVID-19 vaccines. We found that these two heterologous prime-boost regimens were highly effective at raising neutralizing antibody levels against SARS-CoV-2 ancestral strain and Omicron subvariants, BA.1 and BA.5.

Six months after completion of primary series and prior to boosting, the inactivated COVID-19 vaccines BBIBP-CorV and CoronaVac retained neutralizing activity against ancestral SARS-CoV-2 virus, with positivity rates over 50%, but neutralizing responses against Omicron subvariants BA.1 and BA.5 were much lower. Ten months after priming doses, no participants had detectable neutralization against BA.1 or BA.5. Zhang and colleagues found that 4–8 months after a two-dose primary series with inactivated vaccines (BBIBP-CorV or CoronaVac), neutralizing GMTs were still detectable, but were lower compared to 14 days after the second primary series dose [27]. Several other studies have shown attenuation of antibody levels against prototype strain 6 months after primary vaccination regardless of technological platform [10, 11, 29], with even greater declines in antibody levels against Omicron variants [12, 30]. These findings are consistent with our results.

We found that the immunogenicity of heterologous booster doses with both tested vaccines was superior to primary series alone. Thirty days after booster doses, GMTs against prototype strain increased 127.8–428.7-fold, GMTs against BA.1 increased 13.2–37.9-fold, and GMTs against BA.5 increased 10.6–24.7-fold. This is consistent with other studies showing that booster vaccination enhances immune response [22, 26, 28].

The neutralizing response induced by boosting with Ad5-vectored vaccine given as an orally inhaled aerosol (Convidecia Air) was greater than boosting with Convidecia given by intramuscular injection. Wei Chen and colleague found that an Ad5-nCoV booster induced potent neutralizing activity against the ancestral virus and Omicron variants [31], while aerosolized Ad5-nCoV generated the greatest neutralizing antibody responses against the Omicron variant on day 28 after booster vaccination—14.1-fold more than homologous CoronaVac boosting and 2.0-fold more than intramuscular Ad5-nCoV boosting. Compared with intramuscular injectable vaccines, airway mucosal vaccine-elicited IgA and resident memory B and T cells in the respiratory mucosa may provide an effective barrier to infection at these sites. Resident memory B and T cells, which encounter the antigen early and respond more quickly than systemic memory cells, may impede viral replication and reduce viral shedding and transmission. One study showed that an aerosolized Ad5-nCoV booster produced a greater IFNγ T-cell response at 5.0-fold that of intramuscular Ad5-nCoV [31]. These results suggest that for boosting, oral inhalation enhances the nAb responses of aerosolized Ad5-vectored vaccine compared with intramuscular injection.

The kinetics of GMTs against ancestral virus, BA.1, and BA.5 that our study found suggested that GMTs peaked about one month after a booster dose and then declined over the next 5 months, staying always higher than GMTs 6 months after priming doses. A phase 2/3 trial by Moderna of their bivalent (ancestral and Beta variant) vaccine, mRNA-1273.211, given as a first booster 9 months after primary vaccination with mRNA-1273 showed that GMTs against the ancestral SARS-CoV-2 strain with the D614G mutation and Beta, Delta, and Omicron variants were lower 6 months post boosting compared with 1 month post boosting [32]. Among older adults, Vanshylla and colleagues found that neutralizing titers 3.5 months after BNT162b2 booster doses had decreased by 2.7-fold against ancestral virus, 2.3-fold against Delta variant, and 3.0-fold against Omicron variant [33]. A vaccine effectiveness study found that effectiveness of three doses of mRNA-1273 against infection with Delta or Omicron variants began to wane at about 2 months [34].

Omicron subvariants showed substantial resistance to infection-induced and vaccine-induced serum neutralizing activity, regardless of technical platform of the vaccines. Using a pseudovirus assay, Gao and colleagues found that in individuals vaccinated with either three doses of inactivated virus vaccines (BBIBP-CorV or CoronaVac), three doses of the protein-subunit vaccine ZF2001, or two doses of CoronaVac boosted by ZF2001, neutralizing antibody titers against BA.4/5 were 10.8 to 31.6 times lower than titers against the ancestral strain, and 2.1 to 2.6 times lower than titers against BA.2 [35]. Planas and colleagues estimated that after boosting, the duration of neutralization was markedly shorter against BA.5 than against D614G mutation strain (5.5 months vs 11.5 months) [36]. Using a pseudovirus neutralization assay, Ma and colleagues showed that people with BA.1 breakthrough infections had 2.4-times lower neutralizing titers against BA.1 compared with D614G-mutated variant, and people with BA.2 breakthrough infections had 2.3-times lower neutralizing titers against BA.2 compared with D614G-mutated variant [37]. Although differences in neutralization may be due to differences in laboratory methods between pseudovirus and live virus assays, these findings all indicate that neutralizing antibody titers against Omicron subvariants BA.4/5 are significantly lower than corresponding titers against the SARS-CoV-2 prototype isolate, indicating substantial immune escape for Omicron subvariants.

Although neutralizing activity does not equal protection from infection, Khoury and colleagues [38, 39] found that neutralizing antibody titers were strongly correlated with vaccine effectiveness against symptomatic and severe COVID-19, and the higher the ratio of neutralizing antibodies generated after vaccination compared with convalescent levels, the higher the protective rate of the vaccine. According to their correlates model curve, neutralizing antibody levels against ancestral virus of any group in our study was comparable with or higher than convalescent and could yield over 80% vaccine effectiveness against symptomatic illness and over 90% vaccine effectiveness against severe COVID-19 caused by the ancestral virus. It is noteworthy that neutralizing antibody levels against Omicron subvariants BA.1 or BA.5 were 40% above nAb levels of convalescent sera, implying that vaccines can elicit about 60% vaccine effectiveness against symptomatic illness and over 85% vaccine effectiveness against severe COVID-19 caused by Omicron subvariants BA.1 or BA.5.

Strengths of this study are that we studied booster immunization of two distinct routes of booster dose administration (injected and inhaled) and evaluated neutralizing antibodies levels against the ancestral strain and Omicron subvariants. Furthermore, we predicted vaccine effectiveness by comparing Nab responses raised by natural infection and vaccines. Our study has several limitations. First, we only assessed live virus neutralizing antibody levels and did not test individual antigens or cellular immune responses, which play an important role in immunity to the SARS-CoV-2 virus. Second, due to the limited sample size, our study could not conduct subgroup analyses by comorbidity. Third, our study was conducted only in participants aged 18–59 which precludes making conclusions about immune responses in the elderly or in children. Fourth, we did not obtain mucosal samples and therefore cannot address directly mucosal immunity.

## Conclusions

In conclusion, neutralizing activity against the Omicron BA.1 subvariants or BA.5 raised by inactivated vaccines was minimally detectable or undetectable 6 months after priming vaccination. Heterologous prime-boost vaccination with injectable Convidecia or aerosolized Convidecia was immunogenic against not only the SARS-CoV-2 prototype strain but also against Omicron subvariants BA.1 and BA.5. Our study adds to the evidence supporting the current immunization strategies of heterologous boosting in populations primed with inactivated COVID-19 vaccines in China.

## Supplementary Information

**Additional file 1: Table S1. **Immune responses against ancestral virus, BA.1 and BA.5of prime-booster groups by gender.** Table S2. **Immune responses against ancestralvirus, BA.1 and BA.5 of prime-booster groups by Body Mass Index group.

## Data Availability

The data analyzed are not publicly available as they contain personal information. Data with personally identifiable information are available upon request.

## Abbreviations

BMI: Body mass index
COVID-19: Coronavirus disease of 2019
SARS-CoV-2: Severe acute respiratory syndrome coronavirus 2
