# Supplementary material for: Neutralizing antibody levels associated with injectable and aerosolized Ad5-nCoV boosters and BA.2 infection
Source: BMC Med. 2023 Jul 3;21:233. doi: 10.1186/s12916-023-02942-3 (PMC10318644; doi:10.1186/s12916-023-02942-3)
Supplement: Supplementary file 1 — Additional file 1: Table S1. Immune responses against ancestral virus, BA.1 and BA.5of prime-booster groups by gender. Table S2. Immune responses against ancestralvirus, BA.1 and BA.5 of prime-booster groups by Body Mass Index group. [file 12916_2023_2942_MOESM1_ESM.docx]

**Additional File 1**

**Table S1.** Immune responses against ancestral virus, BA.1 and BA.5 of prime-booster groups by gender.

**Table S2.** Immune responses against ancestral virus, BA.1 and BA.5 of prime-booster groups by Body Mass Index group.

**Table S1. Immune responses against ancestral virus, BA.1 and BA.5 of prime-booster groups by gender.**

| Gender | ^a^ Group | Sample collection time |  | Ancestral |  |  | BA.1 |  |  | BA.5 |  |
| --- | --- | --- | --- | --- | --- | --- | --- | --- | --- | --- | --- |
|  |  |  | n | Positive rate (%) | Geometric mean titers (1:) GMT (95%CI) | n | Positive rate (%) | Geometric mean titers (1:) GMT (95%CI) | n | Positive rate (%) | Geometric mean titers (1:) GMT (95%CI) |
| Male | A | 0 day | 47 | 53.19 | 3.64 (3.01, 4.42) | 22 | 0 | 2 | 22 | 0 | 2 |
|  |  | 1 month | 43 | 100 | 692.95 (535.17, 897.25) | 22 | 100 | 32.69 (22.26, 48.02) | 22 | 95.45 | 25.35 (16.7, 38.49) |
|  |  | 6 months | 23 | 100 | 74.07 (45.78, 119.86) | 23 | 86.96 | 14.92 (9.41, 23.65) | 23 | 56.52 | 5.45 (3.41, 8.7) |
|  | B | 0 day | 2 | 0 | 2 | 2 | 0 | 2 | 2 | 0 | 2 |
|  |  | 1 month | 3 | 100 | 845.29 (143.89, 4966) | 3 | 100 | 80.63 (1.51, 4300) | 3 | 100 | 20.97 (0.15, 2977) |
|  |  | 6 months | 3 | 100 | 465.18 (117.02, 1849) | 3 | 100 | 92.3 (1.9, 4478) | 3 | 100 | 18.32 (0.95, 351.5) |
|  | C | 0 day | 14 | 71.43 | 4.82 (3.26, 7.12) | 14 | 64.29 | 3.76 (2.69, 5.26) | 14 | 7.14 | 2.1 (1.89, 2.34) |
|  |  | 1 month | 14 | 100 | 1467.26 (801.71, 2685) | 14 | 100 | 119.9 (59.53, 241.48) | 14 | 100 | 61.71 (31.13, 122.35) |
|  |  | 6 months | 14 | 100 | 1317.79 (613.75, 2829) | 14 | 100 | 49.78 (29.57, 83.8) | 14 | 100 | 21.2 (11.07, 40.59) |
| Female | A | 0 day | 54 | 70.37 | 5.66 (4.42, 7.24) ^b^ | 25 | 8 | 2.23 (1.91, 2.62) | 25 | 4 | 2.09 (1.91, 2.29) |
|  |  | 1 month | 51 | 100 | 513.98 (410.15, 644.1) | 25 | 100 | 24.26 (16.91, 34.82) | 25 | 100 | 18.78 (14.37, 24.53) |
|  |  | 6 months | 22 | 100 | 78.8 (49.81, 124.67) | 22 | 86.36 | 10.44 (6.44, 16.9) | 22 | 50 | 3.56 (2.51, 5.06) |
|  | B | 0 day | 2 | 50 | 3.46 (0, 3722) | 2 | 0 | 2 | 2 | 0 | 2 |
|  |  | 1 month | 9 | 100 | 1242.36 (534.89, 2886) | 9 | 88.89 | 74.23 (22.18, 248.44) | 9 | 100 | 54.55 (17.6, 169.01) |
|  |  | 6 months | 9 | 100 | 456.48 (330.18, 631.1) | 9 | 100 | 74.23 (31.99, 172.21) | 9 | 100 | 17.18 (10.34, 28.56) |
|  | C | 0 day | 9 | 77.78 | 5.51 (2.64, 11.51) | 9 | 66.67 | 3.32 (2.44, 4.52) | 9 | 11.11 | 2.16 (1.81, 2.58) |
|  |  | 1 month | 9 | 100 | 861.4 (271.22, 2736) | 9 | 100 | 131.39 (43.2, 399.61) | 9 | 100 | 40.61 (15.79, 104.48) |
|  |  | 6 months | 7 | 100 | 905.22 (311.3, 2632) | 7 | 100 | 36.54 (10.39, 128.52) | 7 | 85.71 | 10.69 (4.19, 27.29) |

^a^ Group A: BBIBP-CorV+Convidecia; Group B : BBIBP-CorV+ aerosolised Convidecia; Group C : CoronaVac+ aerosolised Convidecia.

^b^ Comparison between gender groups *P*<0.05.

**Table S2. Immune responses against ancestral virus, BA.1 and BA.5 of prime-booster groups by Body Mass Index group.**

| Gender | ^a^ Group | Sample collection time |  | Ancestral |  |  | BA.1 |  |  | BA.5 |  |
| --- | --- | --- | --- | --- | --- | --- | --- | --- | --- | --- | --- |
|  |  |  | n | Positive rate (%) | Geometric mean titers (1:) GMT (95%CI) | n | Positive rate (%) | Geometric mean titers (1:) GMT (95%CI) | n | Positive rate (%) | Geometric mean titers (1:) GMT (95%CI) |
| Normal | A | 0 day | 66 | 62.12 | 4.63 (3.78, 5.67) | 32 | 3.13 | 2.09 (1.91, 2.28) | 32 | 3.13 | 2.07 (1.93, 2.22) |
|  |  | 1 month | 61 | 100 | 585.43 (474.84, 721.76) | 32 | 100 | 27.36 (19.76, 37.9) | 32 | 100 | 23.27 (17.51, 30.91) |
|  |  | 6 months | 28 | 100 | 71.73 (47.17, 109.08) | 28 | 85.71 | 12.5 (8.17, 19.11) | 28 | 60.71 | 4.98 (3.4, 7.3) |
|  | B | 0 day | 4 | 25.00 | 2.63 (1.1, 6.31) | 4 | 0 | 2 | 4 | 0 | 2 |
|  |  | 1 month | 12 | 100 | 1128.33 (598.32, 2127.83) | 12 | 91.67 | 75.78 (29.13, 197.11) | 12 | 100 | 42.95 (15.78, 116.93) |
|  |  | 6 months | 12 | 100 | 458.64 (348.89, 602.93) | 12 | 100 | 78.38 (37.72, 162.9) | 12 | 100 | 17.46 (10.78, 28.27) |
|  | C | 0 day | 11 | 72.73 | 4.76 (3.01, 7.52) | 11 | 72.73 | 3.53 (2.66, 4.67) | 11 | 9.09 | 2.13 (1.85, 2.45) |
|  |  | 1 month | 11 | 100 | 1543.29 (729.64, 3264) | 11 | 100 | 194.99 (80.89, 470.03) | 11 | 100 | 74.97 (36.55, 153.77) |
|  |  | 6 months | 10 | 100 | 2121.66 (1483, 3034) | 10 | 100 | 80.67 (52.24, 124.58) | 10 | 100 | 27.25 (13.05, 56.89) |
| Overweight or obese | A | 0 day | 35 | 62.86 | 4.57 (3.42, 6.09) | 15 | 6.67 | 2.19 (1.8, 2.67) | 15 | 0 | 2 (2, 2) |
|  |  | 1 month | 33 | 100 | 596.4 (439.35, 809.6) | 15 | 100 | 29.07 (18.26, 46.3) | 15 | 93.33 | 18.46 (11.57, 29.46) |
|  |  | 6 months | 17 | 100 | 84.62 (49.1, 145.83) | 17 | 88.24 | 12.58 (7.19, 21.99) | 17 | 41.18 | 3.65 (2.27, 5.86) |
|  | B | 0 day | 0 | —— | —— | 0 | —— | —— | 0 | —— | —— |
|  |  | 1 month | 0 | —— | —— | 0 | —— | —— | 0 | —— | —— |
|  |  | 6 months | 0 | —— | —— | 0 | —— | —— | 0 | —— | —— |
|  | C | 0 day | 12 | 75 | 5.39 (3.07, 9.47) | 12 | 58.33 | 3.63 (2.48, 5.33) | 12 | 8.33 | 2.12 (1.87, 2.41) |
|  |  | 1 month | 12 | 100 | 939.54 (398.59, 2215) | 12 | 100 | 82.23 (39.67, 170.47) | 12 | 100 | 37.73 (17.05, 83.45) |
|  |  | 6 months | 11 | 100 | 673.03 (247.01, 1834) ^b^ | 11 | 100 | 26.36 (12.53, 55.45) ^b^ | 11 | 90.91 | 10.91 (5.41, 22) |

^a^ Group A: BBIBP-CorV+Convidecia; Group B : BBIBP-CorV+ aerosolised Convidecia; Group C : CoronaVac+ aerosolised Convidecia.

^b^ Comparison between BMI groups *P*<0.05.
